# Maternal near-miss and the risk of adverse perinatal outcomes: a prospective cohort study in selected public hospitals of Addis Ababa, Ethiopia

**DOI:** 10.1186/s12884-018-1983-y

**Published:** 2018-08-22

**Authors:** Ewnetu Firdawek Liyew, Alemayehu Worku Yalew, Mesganaw Fantahun Afework, Birgitta Essén

**Affiliations:** 1 Department of Nursing, College of Medicine and Health Sciences, Arba Minch University, Arba Minch, Ethiopia; 2 Department of Preventive Medicine, School of Public Health, Addis Ababa University, Addis Ababa, Ethiopia; 3 Department of Reproductive Health and Health Service Management, School of Public Health, Addis Ababa University, Addis Ababa, Ethiopia; 4 Department of Women’s and Children’s Health, International Maternal and Child Health, Uppsala University, Uppsala, Sweden

**Keywords:** Maternal near-miss, Adverse perinatal outcomes, Prospective cohort study, Public hospitals, Addis Ababa, Ethiopia

## Abstract

**Background:**

Presence of maternal near-miss conditions in women is strongly associated with the occurrence of adverse perinatal outcomes, but not well-understood in low-income countries. The study aimed to ascertain the effect of maternal near-miss on the risk of adverse perinatal outcomes in Ethiopia.

**Methods:**

A prospective cohort study was conducted in five public hospitals of Addis Ababa, Ethiopia. Women admitted from May 1, 2015 to April 30, 2016 were recruited for the study. We followed a total of 828 women admitted for delivery or treatment of pregnancy-related complications along with their singleton newborn babies. Maternal near-miss was the primary exposure and was ascertained using the World Health Organization criteria. Women who delivered without complications were taken as the non-exposed groups. The main outcome was adverse perinatal outcomes. Data on maternal near-miss and perinatal outcomes were abstracted from medical records of the participants. Exposed and non-exposed women were interviewed by well-trained data collectors to obtain information about potential confounding factors. Logistic regressions were performed using Stata version 13.0 to determine the adjusted odds of adverse perinatal outcomes.

**Results:**

A total of 207 women with maternal near-miss and 621 women with uncomplicated delivery were included in the study. After adjusting for potential confounders, women with maternal near-miss condition had more than five-fold increased odds of adverse perinatal outcomes compared to women who delivered without any complications (AOR = 5.69: 95% CI; 3.69–8.76). Other risk factors that were independently associated with adverse perinatal outcomes include: rural residence, history of prior stillbirth and primary educational level.

**Conclusions:**

Presence of maternal near-miss in women is an independent risk factor for adverse perinatal outcomes. Hence, interventions rendered at improvement in maternal health of Ethiopia can lead to an improvement in perinatal outcomes.

## Background

Maternal near-miss is defined by the World Health Organization (WHO) as ‘a women who nearly died but survived a complication during pregnancy, childbirth or within 42 days of termination of pregnancy’ [1].

Several studies have shown that the presence of maternal near-miss conditions in women is strongly associated with the occurrence of adverse perinatal outcomes such as stillbirth, preterm birth, low birth weight, early neonatal mortality, birth asphyxia, and admission to a Neonatal Intensive Care Unit (NICU) [2–7]. For instance, a study by Souza et al. in their multi- country study in eight Latin American countries highlighted that the occurrence of maternal near-miss in women is associated with low birth weight, stillbirth, admission to neonatal ICU and neonatal mortality [7]. Another study from Brazil reported that fetal and neonatal deaths, low birth weight, severe birth asphyxia and prematurity were higher among women with maternal near-miss compared to women who delivered without complications [4, 6]. Similarly, a study from Nigeria reported a four-fold risk of stillbirth and a three-fold risk of low birth weight infant among women with maternal near-miss conditions compared to women who delivered without complications [5].

Many previous studies on the association between maternal near-miss and adverse perinatal outcomes were either cross-sectional or case-control which are subjected to information bias [2, 5, 7]. Majority of the studies also used hospital records to abstract potential maternal characteristics which leads to lack of data on important confounding variables [4, 6]. These confounders may be alternative explanations for an observed association between exposure and outcome variables. Thus, it was not clear whether the adverse perinatal outcomes were due to confounding or because of maternal near-miss [4, 6]. Up-to-date information on the effect of maternal near-miss on the risk of adverse perinatal outcomes is important to know the area of interventions that help to improve perinatal health of the country (Ethiopia). Nevertheless, studies that quantify the effect of maternal near-miss on adverse perinatal outcomes are rare in Ethiopia. Hence, the findings of the current study are important to fill the knowledge gap, and it may provide reliable evidence for policy makers, programmers and health practitioners to improve perinatal health of Ethiopia.

## Methods

### Study settings, design and period

A prospective cohort study was conducted in five public hospitals of Addis Ababa, capital of Ethiopia, from May 1, 2015 to April 30, 2016. The selected hospitals are the major referral hospitals in Ethiopia and provide specialized care both for the mother and neonate. All hospitals have obstetric and neonatal ICU and are responsible for a total of 29,697 live birth deliveries per year. The details of settings with location map have been described elsewhere [8].

### Cohort selection, recruitment and exclusions

Women who developed maternal near-miss were the exposure group. Hence, all women admitted for delivery to the participating hospitals during the study period and fulfilled at least one of the WHO criteria were included as exposed group [1]. Women who delivered without any complications were enrolled as non-exposed group. The controls were selected based on the age-interval category and delivered on the same day of the near-miss event. The details of non-exposed (control) selection has been described elsewhere [9]. The study excluded any women with maternal near-miss that was admitted to the participating hospital for the reason of abortion or ectopic pregnancy as this may not result in viable fetus to assess perinatal outcomes. In addition, those women with maternal near-miss who delivered at another facility (outside the included hospitals) were also excluded as it was difficult to know the perinatal outcomes. We have also excluded women with multiple pregnancies. A total of 31 women were excluded from the study.

### Outcome measure

The primary outcome of interest was adverse perinatal outcomes and was categorized as presence or absence of it. Adverse perinatal outcomes were defined as the presence of either of the following: stillbirth, low birth weight, preterm birth, admission to neonatal ICU and first minute birth asphyxia. Stillbirth was defined as a newborn with no signs of life at or after 28 completed weeks of pregnancy. Low birth weight was defined as a newborn weight below 2500 g. Preterm birth is a baby born alive before 37 completed weeks of gestation but after 28 weeks of gestation. Gestational age was determined on the basis of last menstrual period and ultrasound measures were taken when prediction by last menstrual period was not possible. In order to grade the severity of perinatal asphyxia in newborn, Apgar score was used. The score below 7 at the first minute of life were considered as having first minute birth asphyxia.

### Potential confounders

The following variables were taken as potential confounders: (1) socio-economic and demographic characteristics of the women such as age, educational level, marital status, monthly income, (2) reproductive health and obstetric history of the women such as ANC status, number of children, history of stillbirth, early marriage, (3) pre-existing medical conditions such as previous chronic hypertension, previous anemia and history of cardiac problems. The details of main confounders and their measure have been described elsewhere [9].

### Sample size determination

The sample size was estimated using Epi Info 7 software using sample size determination for cohort studies. The parameters that were used to estimate the sample size were: confidence level of 95%, power of 80%, exposed to non-exposed ratio of 1:3, expected prevalence of outcome in non-exposed group 6%, and prevalence of outcome in exposed group to be 22.2%. It was estimated based on one study in Nigeria taking prevalence of birth asphyxia among exposed and non-exposed women to maternal near-miss [5]. Adding a 10% loss rate, the final sample size required for the study were 55 exposed and 165 non-exposed women; a total of 220 women. However, the current study was part of a larger study which required larger sample size. Thus, all available participants were considered in the current analysis to increase the power of the study.

### Data collection

Information on still/live birth, birth weight, gestational age, Apgar score at 1st minute and admission to neonatal ICU were extracted from the medical records of both exposed and non-exposed women. The records were made during childbirth by health care professionals working in the delivery ward. At the end of childbirth, well-trained nurses and midwives extracted perinatal information for singleton babies using the data abstraction tool adapted from WHO [1]. Maternal near-miss data were also abstracted from medical record of the participants using the WHO data abstraction tool [1]. To know other potential confounders of adverse perinatal outcomes, both exposed and non-exposed women were interviewed using pre-tested structured questionnaires. The participants were interviewed when they became healthy near to their discharge time. The details of questionnaire preparation and maternal near-miss assessment were explained elsewhere [8, 9].

### Data analysis

The data were entered using Epi Info 7 software and analyzed using Stata version 13.0. Cleaning of the data was performed prior to analysis. To see whether there is a statistically significant difference between exposed and non-exposed women with regard to selected categorical variables, chi-square tests were performed. Continuous variables were summarized using the median and Mann-Whitney U test was used for comparison between groups. The statistical significance was set at *p* <  0.05. In order to know the crude association between maternal near-miss and adverse perinatal outcomes, crude odds ratio (COR) of adverse perinatal outcomes with 95% confidence interval (CI) were calculated among exposed and non-exposed women. In addition, *p-*value and crude odds ratio with 95% CI were calculated for each potential confounding variable to evaluate the crude association between potential risk factors and adverse perinatal outcomes. The number and proportion of the outcome variable with regard to exposure status were also calculated. Those variables with *p* <  0.2 from the bivariate analysis were considered for binary logistic regression.

Logistic regression analysis was performed to see the effect of maternal near-miss on adverse perinatal outcomes while controlling for potential confounders. Adjusted odds ratios (AOR) with 95% CI were calculated for each independent variable to see the adjusted association between exposure variables and adverse perinatal outcomes.

Model fitness was assessed using Hosmer–Lemeshow goodness-of-fit tests. Poor fit was indicated by a significance value less than 0.05. Because the significance value of the calculated model in the current analysis was greater than 0.05, there was insufficient evidence of poor model fit.

To check for the presence of multicollinearity among exposure variables, Stata’s estat vif command was used to calculate the variance inflation factors (VIF) for each exposure variable. Possible multicollinearity was suggested if the largest VIF is greater than 10. As all the calculated VIF of each exposure variable in our study was less than 10, no possible multicollinearity was observed.

## Results

During the one-year period, a total of 238 women with maternal near-miss were observed in the five participating hospitals. However, 22 of them were at less than 28 weeks of gestation and 9 cases gave birth outside the participating hospitals. Hence, we finally considered and followed 207 women with maternal near-miss (exposed women) and 621 corresponding non-exposed women (uncomplicated delivery group) as a cohort for final analysis.

Women exposed to maternal near-miss tended to be illiterate (*p* <  0.001), unmarried (*p* = 0.021), had less monthly income (*p* = 0.003) and more likely to reside in the rural area (*p* <  0.001) compared to non-exposed women. The two groups did not significantly differ in terms of age (*p* = 0.673), religion (*p* = 0.676) and ethnicity (*p* = 0.054) (Table 1).

**Table 1:** Distribution of selected variables among women with near-miss and uncomplicated delivery women in selected five public hospitals, Addis Ababa, Ethiopia, May 1, 2015 to April 30, 2016

	Near-miss group (*n* = 207)	Uncomplicated delivery group (*n* = 621)	*P-value
Characteristics	n (%)	n (%)	
Educational level			
Illiterate	57 (29.2)	75 (12.2)	**< 0.001**
Primary	62 (31.8)	201 (32.7)	0.323
Secondary	55 (28.2)	249 (40.5)	0.847
Higher	21 (10.8)	90(14.6)	
Place of residence			
Urban	153 (73.9)	608 (97.9)	
Rural	54 (26.1)	13 (2.1)	**< 0.001**
Marital status			
Married	192 (92.8)	600 (96.6)	
Never married	15 (7.2)	21 (3.4)	**0.021**
Monthly income			
> 68 USD	76 (36.7)	108 (17.4)	**0.003**
68–181 USD	73 (35.3)	358 (57.6)	**0.002**
> 181 USD	58 (28.0)	155 (25.0)	
Received ANC			
Yes	177 (85.5)	611 (98.4)	
No	30 (14.5)	10 (1.6)	**< 0.001**
Number of children			
0–2	164 (79.2)	507 (81.6)	0.855
3–4	32 (15.5)	103 (16.6)	
> 5	11 (5.3)	11 (1.8)	**0.013**
Undergone FGC			
Yes	129 (64.5)	366 (59.4)	0.201
No	71 (35.5)	250 (40.6)	
History of stillbirth			
Yes	20 (9.7)	20 (3.2)	**< 0.001**
No	187 (90.3)	601 (96.8)	
Early marriage			
Yes	41 (21.5)	90 (15.1)	**0.041**
No	150 (78.5)	506 (84.9)	
Previous hypertension			
Yes	54 (26.1)	16 (2.6)	**< 0.001**
No	153 (73.9)	605 (97.4)	
Previous anemia			
Yes	70 (33.8)	63 (10.1)	**< 0.001**
No	137 (66.2)	558 (89.9)	
History of cardiac problems			
Yes	11 (5.3)	5 (0.8)	**< 0.001**
No	196 (94.7)	616 (99.2)	

*Chi-square test was used to obtain the *p*-value

Bold data are those which are significant

Compared to non-exposed women, majority of the exposed women were less likely to receive ANC (*p* < 0.001) and more likely to have had more than five children (*p* = 0.013), a history of stillbirth (*p* < 0.001) and married early (*p* = 0.041). There was no statistically significant difference among the two groups with regard to the female genital cutting (FGC) status of the women (*p* = 0.201) (Table 1).

Women with maternal near-miss were more likely to report a previous history of chronic hypertension and cardiac problems (both *p* < 0.001) (Table 1).

Table 2 is about comparison of the adverse perinatal outcomes among exposed and non-exposed women. From a total of 828 women delivered in the participating hospitals, 36.6% (95% CI:33.4 – 39.9%) of them end up in a wide range of adverse perinatal outcomes such as stillbirth, preterm birth, low birth weight infant, birth asphyxia and admission to neonatal ICU. The prevalence of adverse perinatal outcomes was significantly higher among women who were exposed to maternal-near miss compared to the non-exposed women, 72.9% (95% CI: 66.5 – 78.5%) versus 24.5% (95% CI:21.3 – 28%) respectively, *p* < 0.001. Babies born from women with maternal near-miss were more likely to be stillborn (*p* < 0.001), preterm (*p* < 0.001), of lower birth weight (*p* < 0.001), admitted to neonatal ICU (*p* < 0.001) and tended to have had a birth asphyxia in the first minute (*p* < 0.001) (Table 2).

**Table 2:** Prevalence of adverse perinatal outcomes among women with near-miss and uncomplicated delivery women in selected five public hospitals, Addis Ababa, Ethiopia, May 1, 2015 to April 30, 2016

	Groups				*P-value	COR (95% CI)
	Uncomplicated delivery (n = 621)		Near-miss (n = 207)			
Outcome variables	No	%	No	%		
Adverse perinatal outcomes	152	24.5	151	72.9	< 0.001	**8.32 (5.82–11.89)**
Stillbirth	24	3.9	61	29.5	< 0.001	**10.39 (6.27–17.23)**
Preterm birth	48	7.7	84	40.6	< 0.001	**8.15 (5.44–12.22)**
Low Birth weight	50	8.1	82	39.6	< 0.001	**7.49 (5.02–11.19)**
Asphyxia at 1 min	73	11.8	119	57.5	< 0.001	**10.15 (7.03–14.67)**
Admitted to **NICU	52	8.4	61	29.5	< 0.001	**4.57 (3.03–6.9)**

*Chi-square test was used to obtain the *p*-value

**NICU stands for Neonatal Intensive Care Unit

Bold data are those which are significant and their significance is indicated by the *P*-values expressed at the left of each CORs

A statistically significant difference in hospital stay was also observed between the two groups. Women with maternal near-miss were more likely to have a longer median hospital stay of 6 days compared to non-exposed women with a median hospital stay of 1 day (*p* < 0.001).

### Risk factors of adverse perinatal outcomes

After adjustment for potential confounders such as educational level, place of residence, monthly income, ANC status, history of stillbirth, and presence of previous chronic hypertension, anemia, and cardiac problems in a logistic regression analysis, the association between maternal near-miss and adverse perinatal outcomes remained significant. The odds of developing adverse perinatal outcomes among women who developed maternal near-miss was more than five times higher than among women with no maternal near-miss (AOR = 5.69: 95% CI;3.69–8.76) (Table 3).

**Table 3:** Maternal near-miss and odds of adverse perinatal outcomes in relation to other confounding variables in selected five public hospitals, Addis Ababa, Ethiopia, May 1, 2015 to April 30, 2016

	Adverse perinatal outcomes	
	COR (95% CI)	*AOR (95% CI)
Characteristics		
Maternal near-miss		
Yes	**8.32(5.82–11.89)**	**5.69(3.69–8.76)**
No	1	1
Educational level		
Illiterate	**3.11(1.79–5.04)**	1.56(0.80–3.04)
Primary	**2.03(1.24–3.35)**	**1.89(1.07–3.34)**
Secondary	1.37(0.83–2.26)	1.45(0.83–2.52)
Higher	1	1
Place of residence		
Rural	**7.74(4.21–14.21)**	**2.16(1.03–4.53)**
Urban	1	1
Monthly income		
< 68 USD	**1.89(1.26–2.82)**	1.21(0.73–1.98)
68 to 181 USD	.77(.55–1.09)	0.87(0.58–1.32)
> 181 USD	1	1
Received ANC		
Yes	1	1
No	**5.66(2.73–11.75)**	1.86(0.79–4.41)
History of stillbirth		
Yes	**3.43(1.76–6.67)**	**2.39(1.12–5.10)**
No	1	1
Previous hypertension		
Yes	**3.49(2.09–5.82)**	1.24(0.66–2.32)
No	1	1
Previous anemia		
Yes	**2.05(1.41–2.98)**	0.98(0.61–1.57)
No	1	1
Previous cardiac problems		
Yes	**2.95(1.06–8.21)**	1.29(0.36–4.56)
No	1	1

*Single model was used to produce the AORs

*Adjusted for the eight variables shown in the table

Bold data are those which are significant

Educational level, place of residence and prior stillbirth delivery also remained independently associated with adverse perinatal outcomes in logistic regression analysis. The effect of maternal near-miss on adverse perinatal outcome was exacerbated when the women had a primary level of education (AOR = 1.89: 95% CI; 1.07–3.34), resided in rural areas, (AOR = 2.16: 95% CI; 1.03–4.53) and had a history of stillbirth (AOR = 2.39; 95% CI; 1.12–5.10) (Table 3).

## Discussion

The main finding of the study is that the presence of maternal near-miss is a risk factor for adverse perinatal outcomes independent of educational level, place of residence, monthly income, ANC follow-up, history of stillbirth, and presence of previous hypertension, anemia and cardiac problems.

Higher risk of adverse perinatal outcomes such as stillbirth, low birth weight, preterm birth, admission to neonatal ICU, birth asphyxia and early neonatal mortality were also observed among maternal near-miss women in studies conducted in Nigeria, Brazil and other 8 Latin American countries [5–7]. The Nigerian study used case-control design and higher risk of poor perinatal outcomes such as stillbirth and low birth weight infants were reported among women with maternal near-miss compared to the control group [5]. Serious maternal complications generally will lead to interventions which sometimes may also reduce gestational age and thus lead to preterm birth and low birth weight. Unlike the present study, information on potential confounders has been obtained from the medical records in other studies [6, 7]. Hence, the previous studies might be subjected to information bias due to incompleteness and poor quality of secondary data at the health facility. A woman with maternal near-miss could develop severe conditions which include eclampsia, anemia, ante-partum hemorrhage and placenta praevia among others. These severe conditions can affect the fetus, for example, through placental insufficiency leading to intrauterine growth restriction (IUGR). Preeclampsia, for instance, is associated with IUGR and prematurity [10]. IUGR is associated with distress and asphyxia and is the second cause of perinatal deaths [11, 12]. Studies also documented that preterm babies are immature and more likely to be stillbirth, smaller, require an ICU and are a major cause of neonatal mortality [13, 14]. Reduction in adverse perinatal outcomes among pregnant women might be achieved through provision of proper ANC to early diagnose placental insufficiency. The information is also important for health care providers to conduct different tests that detect placental insufficiency. It also highlights the importance of treating the underlying maternal conditions such as high blood pressure and anemia. It further signifies the importance of health education for pregnant women on various issues such as frequent ANC visits and bed rest.

The study also documented that women in rural locations were more likely to experience adverse perinatal outcomes regardless of the near-miss status of the women. This is in agreement with other studies [15, 16]. Studies shown that women residing in rural areas with no access to obstetric care had to travel longer to get routine antenatal care and skilled birth attendance, barriers associated with adverse perinatal outcomes [17, 18]. For instance, various studies reported that higher numbers of low birth weight babies were seen in women who had irregular ANC visits compared to women who had regular ANC checkups [19, 20]. Rural women are also relatively disadvantaged in terms of their socio-economic status which could possibly increase their risk of adverse perinatal outcomes. For example, rural women tend to have a lower educational level and higher rate of poverty compared to urban women [21].

In this study, women who had prior stillbirth in preceding births were at higher risk of having adverse perinatal outcomes than women without a history of stillbirth. Available evidence suggests that women with stillbirth in their prior pregnancy were at higher risk of adverse perinatal outcomes in subsequent pregnancies [22–27]. Another independent risk factor for adverse perinatal outcomes was level of education. Women who had a primary level of education had a higher risk of having adverse perinatal outcomes than those with a higher level of education. Education enhances the health care seeking behavior of the women so that they can effectively utilize maternal health care services when complications happen [28]. A growing body of literature has revealed that lower levels of maternal education were associated with an increased risk of variety of adverse perinatal outcomes [16, 25, 29–31].

The study has several strengths. To our knowledge, this study is the first of its kind in Ethiopia to document the effect of maternal near-miss on adverse perinatal outcomes using a prospective cohort design. The study used the standard WHO criteria to assess maternal near-miss and hence, we ascertained the exposure status. We studied a variety of perinatal outcomes which includes stillbirth, preterm birth, birth weight, birth asphyxia and admission to neonatal ICU. We also collected information on many confounding variables. To see the effect of adverse perinatal outcomes among exposed and non-exposed groups, the effect of other possible determinants of adverse perinatal outcomes were controlled during analysis. Furthermore, adequate training was given to data collectors to obtain data in the same fashion which avoids the presence of bias related to measurements. There was also no loss to follow-up in our study. Although there is a considerable variation in severity among the different perinatal outcomes investigated in our study, we opt to merge these outcomes. This is due to the fact that the sample size was not sufficient to separately investigate the outcomes, thus merging increased the sample size and minimized the role of chance.

Because of logistic and feasibility concerns, the study did not look for some of the important perinatal outcomes such as neonatal mortality among women with maternal near-miss. The short and long term maternal consequences of near-miss events were also not addressed in the current study which calls for the importance of other big studies. Although near-miss includes events within 42 days of termination of pregnancy, we followed women only to the length of hospital stay because of logistic and feasibility concerns. Although the five minutes Apgar score is more sensitive indicator of birth asphyxia, we have considered the one minute Apgar score in our study.

The study was conducted only in public hospitals. Hence, the pattern of adverse perinatal outcomes among the two groups does not represent the larger group in the population. However, the results of the study can be generalized to public hospitals in Addis Ababa, beyond those hospitals included in the current study. Poor perinatal outcomes could be related to poor quality of care around childbirth [32]. However, the study did not explore quality of care domains. Hence, we recommend further studies to better understand challenges to quality of care for women and newborns.

## Conclusions

The study demonstrated that women with maternal near-miss complications during pregnancy and delivery were more likely to have adverse perinatal outcomes. Hence, this suggests that evidence-based interventions rendered at improvement in maternal health of Ethiopia can lead to an improvement in perinatal outcome.

## Abbreviations

ANC: Antenatal care
AOR: Adjusted odds ratio
Apgar: Activity, pulse, grimace, appearance, and respiration
CI: Confidence interval
COR: Crude odds ratio
FGC: Female genital cutting
ICU: Intensive care unit
IUGR: Intrauterine growth restriction
NICU: Neonatal intensive care unit
VIF: Variance inflation factors
WHO: World Health Organization
